# m6AHD: a new framework for identifying abnormal N6-methyladenosine (m6A) in heart diseases based on sequencing features

**DOI:** 10.3389/fgene.2026.1776616

**Published:** 2026-02-25

**Authors:** Jiajie Lu, Yanan Li, Yuxiang Hong, Dongshan Liao, Guanhua Fang

**Affiliations:** 1 Department of Cardiovascular Surgery, Fujian Medical University Union Hospital, Fuzhou, Fujian, China; 2 Heart Center of Fujian Medical University, Fuzhou, Fujian, China; 3 Key Laboratory of Ministry of Education for Gastrointestinal Cancer, School of Basic Medical Sciences, Fujian Medical University, Fuzhou, China; 4 School of Medical Technology and Engineering, Fujian Medical University, Fuzhou, China; 5 Department of Clinical Laboratory, Fujian Medical University Affliated First Quanzhou Hospital, Quanzhou, Fujian, China

**Keywords:** bioinformatics framework, cardiac disease, m6A, machine learning, RNA modification

## Abstract

**Introduction:**

Cardiovascular disease (CVD) is a major threat to health, with high incidence rates and a trend toward younger age groups. RNA modifications are an important component of epigenetics, widely present and indispensable in cells. Increasing evidence suggests that RNA modifications are key regulatory factors involved in cardiac physiological and pathological changes. Understanding the role of RNA modifications in heart-related diseases can help us to identify new drug targets.

**Methods:**

To systematically investigate the role of m6A modification in different cardiac diseases, we integrated m6A epitranscriptome profiles from five cardiac pathological conditions (three drug-induced cardiac toxicity models—Evodiamine, Matrine, and TKI, hypertrophy, and heart calcification) and their control groups to construct the first predictive model for abnormal m6A modification in cardiac diseases. We constructed separate models for upregulated and downregulated modifications under different pathological conditions, performed feature selection and parameter optimization, and validated the performance of our models using an independent test set.

**Results:**

m6AHD demonstrated excellent performance on the independent test set, with AUROC scores ranging from 0.728 to 0.880 across various pathological conditions. Cross-validation across different conditions and model interpretability demonstrated that m6A modifications exhibit similar patterns under different pathological conditions and are potentially regulated by similar factors, providing new clues for identifying targets in cardiovascular diseases at the epitranscriptome level. Furthermore, we validated our findings using a zebrafish model of Evodiamine-induced cardiotoxicity. The experimental results revealed significant morphological defects and a broad downregulation of m6A methyltransferase complex components, confirming the involvement of aberrant m6A machinery in the pathology of cardiotoxicity.

**Discussion:**

m6AHD is the first dedicated framework for predicting multi-condition cardiac m6A dysregulation. Our findings underscore the critical role of m6A homeostasis in cardiomyocyte function and demonstrate that aberrant methylation patterns can serve as reliable indicators of cardiac pathology. This framework provides a robust computational tool for identifying potential therapeutic targets at the epitranscriptome level for cardiovascular diseases.

## 1 Introduction

Over the past decade, RNA modifications have emerged as key regulatory factors in gene expression and RNA metabolism. To date, over 170 distinct types of RNA modifications have been identified in both coding and non-coding RNAs, spanning three domains of life and viruses (Boccaletto et al., 2022). Among these, N6-methyladenine (m6A) is the most common internal modification in mammalian messenger RNA (mRNA) and long non-coding RNA (lncRNA). m6A plays a crucial role in various biological processes, including cell cycle regulation, embryonic development, and apoptosis. It also regulates multiple aspects of RNA metabolism, such as RNA structural remodeling, transcript stability, translation efficiency, nuclear export, and subcellular localization.

In recent years, an increasing number of studies have demonstrated that RNA modifications play a crucial regulatory role in the onset and progression of cardiovascular diseases, particularly in pathological states such as myocardial hypertrophy, fibrosis, and heart failure, where RNA modifications exert their effects by regulating the expression of key genes. In myocardial hypertrophy, experimental data show that cardiomyocytes overexpress the m6A methyltransferase METTL3, leading to high methylation of mRNA associated with myocardial hypertrophy-related genes. This affects myocardial hypertrophy-related signaling pathways, inducing myocardial hypertrophy and pathological remodeling (Kumari et al., 2022). Additionally, non-coding RNAs such as cardiac-hypertrophy-associated piRNA (CHAPIR) can interact with METTL3, inhibiting its methylation activity and regulating the stability of PARP10 mRNA. Imbalances in this process may exacerbate the hypertrophy process (Kumari et al., 2022; Wang et al., 2023). RNA modifications also play a critical role in the development of myocardial fibrosis and heart failure. Studies have shown that after myocardial infarction, the expression of the m6A demethylase FTO is significantly downregulated, leading to hypermethylation of SERCA1 mRNA transcripts, abnormal protein translation, and subsequent intracellular calcium ion imbalance, thereby triggering myocardial infarction and heart failure (Kumari et al., 2022). Additionally, an increase in global m6A modification levels after cardiac injury promotes the activation and proliferation of fibroblasts, accelerating the fibrosis process and resulting in abnormal cardiac structural remodeling (Wang et al., 2023).

The omics technology has promoted the understanding of the function of RNA modifications in physiological and pathological conditions of the heart. High-throughput sequencing methods, such as MeRIP-seq, have enabled the analysis of RNA modifications (such as m5C (Ma J. et al., 2022), m6A, m7G (Wang et al., 2024) across the entire transcriptome of different cardiac conditions. In parallel with these methods, numerous computational tools have been developed to identify and interpret RNA modification. Several predictors based on sequence features and machine learning, such as SRAMP (Zhou et al., 2016), WHISTLE (Chen K. et al., 2019), Gene2Vec (Zou et al., 2019), TransRM (Liu et al., 2025),and Geo2Vec (Huang et al., 2022), have been proposed for detecting m6A modification sites. Additionally, databases have been constructed to organize m6A modification sites and regions, integrating annotations such as RNA-binding protein (RBP) interactions and predicted regulatory functions. These resources enable researchers to explore the functional relevance of RNA modifications in diseases (Song et al., 2023a), mutation hotspots (Song et al., 2023b) and evolutionary conservation (Song et al., 2021). Furthermore, expression patterns regulated by RNA modifications have been used to develop prognostic models for certain diseases (Zheng et al., 2022).

Although multifunctional m6A prediction tools based on sequence features have been developed to identify potential methylation sites, such as SRAMP (Zhou et al., 2016) and WHISTLE (Chen K. et al., 2019; Huang et al., 2024), these tools are primarily designed to detect static sites under normal physiological conditions. They often lack the specificity required to distinguish abnormal, disease-associated methylation patterns from background signals. In light of the mounting evidence linking m6A dysregulation to cardiovascular pathology, there is an urgent need for a systematic investigation into its role in heart disease. Consequently, a dedicated states must be established in order to reveal post-transcriptomic features that are functionally relevant. In this study, we propose m6AHD (m6A association in heart disease), a novel machine learning-based predictive model designed to identify hypermethylated and hypomethylated m6A sites in five cardiac pathological states. These pathological states include three drug-induced cardiac toxicity models (Evodiamine, Matrine, and TKI), hypertrophy, and heart calcification. By integrating disease cohort and control group MeRIP-seq data, m6AHD aims to uncover disease-specific m6A methylation patterns and provide insights into the mechanistic role of m6A in cardiovascular dysfunction.

## 2 Methods and materials

**FIGURE 1:**
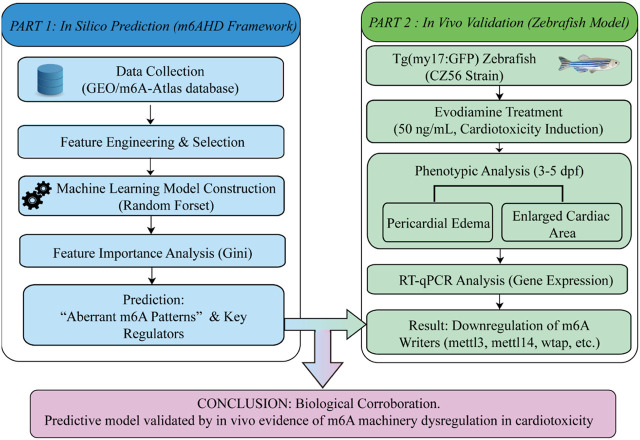
Flow chart of the current study.

The flow chart of the analyses in the current study is shown in Figure 1.

### 2.1 Datasets construction

To predict heart-related diseases using abnormal m6A modification sites, we obtained methylation sites from five heart disease conditions and their corresponding control groups using Methylated RNA Immunoprecipitation Sequencing (MeRIP-seq) from the Gene Expression Omnibus (GEO) (Barre et al., 2013) dataset (Table 1). We also obtained single-base resolution m6A modification sites by m6A-SAC-seq (Hu et al., 2022) through the m6A-Atlas database (Liang et al., 2024) (Supplementary Table S1). In this study, we defined the overlapping sites obtained from MeRIP-seq and single-base resolution sites as true m6A methylation modification sites. We defined abnormal m6A modification as two cases: m6A modification upregulation and m6A modification downregulation. Methylation modification sites present only in the experimental group were defined as m6A upregulation sites, while methylation modification sites present in both the experimental and control groups were defined as their corresponding control group sites. We defined methylation modification sites present only in the control group as m6A downregulated sites and sites that were not methylated in either the experimental or control groups as their corresponding control group sites. In total, we obtained 20 datasets for analysis. In addition, we retrieved datasets for three additional cardiovascular diseases—dilated cardiomyopathy, ischemic cardiomyopathy, and aortic dissection—from the GEO database (Table 1) to serve as independent validation cohorts, thereby demonstrating the generalizability and robustness of our model.

**TABLE 1:** Sequencing results for methylation sites from five heart disease conditions and their corresponding control groups.

GEO	Tissues or cell line	Conditions	Ref
GSE274753	AC16	Evodiamine induced cardiotoxicity	Fang et al. (2024)
		Matrine induced cardiotoxicity	
		Control	
GSE227247	heart valve tissues	Calcified	​
		Non-calcified	
GSE159243	heart	Hypertrophy	Hinger et al. (2021)
		Health	
GSE192913	hiPSC-CMs	TKI-induced cardiotoxicity	Ma Y. et al. (2022)
		Control	
GSE131296	heart tissue	Dilated Cardiomyopathy (DCM)	Berulava et al. (2020)
		Ischemic Cardiomyopathy (ICM)	
		Normal Functioning Heart (NF)	
GSE147028	aorta	Aortic dissection	Zhou et al. (2021)
		Healthy	

The dataset GSE227247 is publicly available at the NCBI Gene Expression Omnibus (https://www.ncbi.nlm.nih.gov/geo/query/acc.cgi?acc=GSE227247).

In our predictions, we utilized the m6A-upregulated sites of each pathological state and their corresponding control sites, m6A downregulated sites with their control group sites to train two separate prediction models. For example, AC16EV_down was trained using evodiamine-induced cardiac toxicity samples, where sites with no methylation (positive sites in the experimental group but not in the control group) and sites with no methylation in both the experimental and control groups (negative sites) were used. AC16EV_up was trained using evodiamine-induced cardiac toxicity samples, where positive sites were those with methylation in the experimental group but not in the control group, and negative sites were those without methylation in both groups. When training the predictor, we first randomly selected 1,000 samples from each of the positive and negative site datasets, with 80% of the samples used for training and the remaining 20% for independent testing. We trained the model using full transcriptomic data, considering both unmodified and methylated sites from exons and introns.

### 2.2 Feature encoding methods

#### 2.2.1 One-hot encoding (OH)

One-hot encoding, a binary encoding method that has gained a high level of recognition within academic discourse, is used to convert nucleotides contained within a biological sequence into a numerical form (Zhou et al., 2016). Research has shown that one-hot encoding is an effective scheme for predicting RNA modification sites. In this encoding scheme, ‘A’, ‘C’, ‘G’, and ‘U’ are represented by binary vectors of (1, 0, 0, 0), (0, 0, 1, 0), (0, 0, 0, 1), and (0, 1, 0, 0), respectively. Accordingly, an RNA sequence with n nucleotides is encoded into a 4 × n dimensional binary vector. To illustrate this point, consider the sequence UACGC, which is converted into binary vectors (0, 0, 0, 1), (1, 0, 0, 0), (0, 1, 0, 0), (0, 0, 1, 0), (0, 1, 0, 0).

#### 2.2.2 Nucleic acid composition (NAC)

In our study, we utilized the frequencies of dinucleotide pairs to encode sequences, represented by a 16-dimensional feature vector that included all combinations from AA to UU. The feature vector $F_{i}$ is expressed as ( $f_{\text{AA}}$ , $f_{AC}$ , $f_{AG}$ , …, $f_{UU}$ ), where *f* denotes the relative frequency of each dinucleotide pair within the *i*th sequence.

#### 2.2.3 Chemical property (CP)

Each RNA nucleotide can be characterised by three distinct features based on its chemical properties (Liu et al., 2020a). C and U possess a single ring structure, while A and G are characterized by a double ring structure. Both A and C contain an amino group, while both G and U are associated with a keto group. During hybridization, A and U form two hydrogen bonds, but triple hydrogen bonds occur in G and C hybridization. Consequently, a nucleotide can be represented by a three-dimensional vector S = ( $x_{i}$ , $y_{i}$ , $z_{i}$ , such as Equation 1):

$$x=\left\{\begin{array}{cc} 1, & s\in \left\{A,G\right\}\\ 0, & s\in \left\{C,U\right\} \end{array}\right.,y=\left\{\begin{array}{cc} 1, & s\in \left\{A,C\right\}\\ 0, & s\in \left\{G,U\right\} \end{array}\right.,z=\left\{\begin{array}{cc} 1, & s\in \left\{A,U\right\}\\ 0, & s\in \left\{C,G\right\} \end{array}\right.$$ (1)

Consequently, the four bases, A, C, G, and U, can be encoded as [1, 1, 1], [0, 1, 0], [1, 0, 0], and [0, 0, 1], respectively.

#### 2.2.4 Electron-ion interaction pseudopotential (EIIP)

Each nucleic acid has a unique electron-ion interaction potential (EIIP) value: A is 0.1260, U is 0.1335, C is 0.1340, and G is 0.0806. Using this method, we can convert an RNA sequence into a numerical vector based on the corresponding EIIP values of each nucleotide. For instance, the sequence “UACG” would be represented as the vector (0.1335, 0.1260,0.1340, 0.0806).

#### 2.2.5 Accumulated nucleotide frequency (ANF)

This method transforms each nucleotide sequence into a series of position-specific features by sequentially evaluating the cumulative frequency of each nucleotide (Chen and Wong, 2020). The feature assigned to the *i*th position is representative of the relative frequency of the nucleotide observed among the first i positions, thereby reflecting the sequential composition dynamics of the sequence.

#### 2.2.6 Pseudo k-tuple nucleotide composition (PseKNC)

In the field of bioinformatics, PseKNC has been widely applied as an encoding method across various domains, including protein, DNA, and RNA prediction (Feng CQ. et al., 2019; Feng et al., 2017; Feng P. et al., 2019; Guo et al., 2014; Pan et al., 2018; Liu et al., 2018; Su et al., 2018). Numerous bioinformatics tools, web-based platforms, and software packages have integrated the PseKNC method into their functional libraries. In this study, we primarily employed the PseDNC-encoding method from the PseKNC framework (Chen et al., 2014).

### 2.3 Algorisms and evaluation

Machine learning algorithms possess powerful data processing and pattern recognition capabilities, rendering them widely applicable in the field of bioinformatics, particularly in the prediction of DNA, RNA, and protein modifications. This study utilized the R program (version 4.4.2) and related packages to train models for the two abnormal m6A modifications in each pathological state, and optimized the models to obtain the final predictors. The sequence length is critical for prediction accuracy. To select the optimal sequence length, we tested 21, 31,41, 51, 61, 71, and 81 nucleotides (nt) centered around the m6A modification sites. In addition to the sequence length, selecting an appropriate feature-encoding method is equally important. We employed six encoding methods and combined all 63 single or combined features for training and testing, ultimately selecting the best feature combination for further optimization.

To determine the most suitable algorithm for model construction, we systematically compared four machine learning algorithms currently popular in bioinformatics research, including Support Vector Machines (SVM, e1071 package (Dimitriadou et al., 2008), Random Forests (RF, randomForest (Liaw and Wiener, 2002),generalized linear models (GLM, stats package (R Core Team R, 2020), and efficient gradient boosting (XGBoost, xgboost package (Chen and Guestrin, 2016). We evaluated the performance of the predictors through independent testing and explored the impact of the parameter selection. The primary performance metric was the area under the receiver operating characteristic curve (AUROC). Additionally, we calculated the accuracy (ACC), sensitivity (Sn), and specificity (Sp) to provide a comprehensive comparison of the algorithm performance (Equation 2). To rigorously evaluate model’s performance on potentially imbalanced datasets, we concurrently employed the Area Under the Precision-Recall Curve (AUPRC). This metric provides a more comprehensive assessment of a classifier’s ability to accurately identify positive samples (true m6A sites) with high precision, complementing AUROC analysis.

$$Sn=\frac{TP}{TP+FN}Sp=\frac{TN}{FP+TN}ACC=\frac{TP+TN}{TP+FP+TN+FN}$$ (2)

### 2.4 Model interpretability via mean decrease gini

To further elucidate the driving mechanisms behind the model’s predictions, we employed the Mean Decrease Gini from a random forest to assess feature importance (Breiman, 2001). This method measures how much each feature reduces node impurity across all splits in the forest. Specifically, for each split using a given feature, the decrease in impurity from the parent node to the child nodes is recorded and summed over all splits. The resulting value reflects the overall contribution of the feature to reducing the uncertainty of the model. A key advantage of this method is its efficiency, as it leverages the existing structure of the random forest without requiring additional computation.

For our predictive model, we used the Mean Decrease Gini to analyze feature importance across different feature extraction methods. By calculating the Mean Decrease Gini value for each feature, we identified the features that contributed most significantly to the model’s classification decisions. This not only enhanced the interpretability of the machine learning model, but also provided biological insights into the most distinctive features of aberrant m6A sites under cardiac pathological conditions.

### 2.5 Animal model and morphological analysis

To visualize cardiac morphology *in vivo*, the transgenic zebrafish line Tg(myl7:GFP) (Catalog ID: CZ56), obtained from the China Zebrafish Resource Center (CZRC), was used in this study. Zebrafish were maintained under standard conditions at 28.5 °C with a 14 h light/10 h dark cycle. Healthy embryos were collected and randomly divided into the control group and the Evodiamine treatment group. At 24 h post-fertilization (hpf), embryos in the treatment group were exposed to Evodiamine at a concentration of 50 ng/mL, while the control group was treated with 0.1% DMSO. The cardiac morphology of zebrafish larvae was observed at 3, 4, and 5 days post-fertilization (dpf) using a stereomicroscope. Pericardial cavity area and cardiac area were quantified to assess the extent of cardiotoxicity. ImageJ software was used for image processing and quantitative analysis.

### 2.6 RT-qPCR

Total RNA was extracted from 30 zebrafish larvae at 4 days post-fertilization (dpf) per group using Trizol reagent (Invitrogen, Carlsbad, CA, United States) method. Subsequently, total RNA was reverse-transcribed into cDNA templates using the Evo M-MLV RT Reaction Mix kit (Accurate Biology, Changsha, China). The primer sequences specific to the m6A modification-related genes, including METTL3, METTL5, METTL14, METTL16, RBM15B, RBM15, VIRMA, ZC3H13, and WTAP, are listed in Table 2. RT-qPCR was performed using the AriaMX real-time PCR system (Agilent, Santa Clara, CA, United States). The thermal cycling conditions comprised an initial denaturation step at 95 °C for 30 s, followed by 40 cycles at 95 °C for 5 s and 60 °C for 30 s. A dissociation curve analysis was performed according to the instrument’s default settings. The relative mRNA expression levels of each targeted gene were normalized to ACTB2 and calculated using the 2^−ΔΔCT^ method.

**TABLE 2:** Sequence of primers used in RT-qPCR.

Primer name	Primer sequence (5′to 3′)
zACTB2-F	CCC​AAA​CCC​AAG​TTC​AGC​CA
zACTB2-R	ACC​CAC​GAT​GGA​TGG​GAA​GA
zMETTL3-F	TAA​GGT​TCA​AGC​GTC​TCA​CC
zMETTL3-R	TCT​CTT​GGC​TCA​CCT​TTT​TGC
zMETTL5-F	ACG​TGT​GCT​CGA​TTG​GAT​CT
zMETTL5-T	ACT​GCA​TGT​CAA​TAC​CCT​GGT​T
zMETTL14-F	CAA​CAA​AAA​CAA​CCC​CGG​CA
zMETTL14-R	TCA​GGC​AGT​GCT​CCT​TAG​TTC
zMETTL16-F	GCG​CTG​AAG​GAA​GAG​TCC​AT
zMETTL16-R	CTC​GAG​TTC​ACT​CCC​TTT​GCT
zVIRMA-F	AAA​GGC​TTC​AGA​CTG​GGC​AA
zVIRMA-R	GTC​AGA​CCT​TCC​GCT​TGT​GTA
zZC3H13-F	CAG​AGA​TGA​ACG​ACG​GGG​AG
zZC3H13-R	GGC​GCT​CTT​TGT​TCT​CGT​TC
zWTAP-F	ACT​CTT​TCG​TAT​CTC​ACA​TTG​GA
zWTAP-R	TGG​TCA​TTC​TGA​TCT​CAG​AGC​C
zRBM15-F	CCG​CAT​ACA​TAG​CAG​AGC​GA
zRBM15-R	CTC​TCT​GGT​CGT​CCT​CTG​GA
zRBM15B-F	ATC​AGC​CCA​AAC​TGG​ACG​AG
zRBM15B-R	GGC​CAG​GAG​GAC​TGC​ATA​TC

## 3 Results

### 3.1 Data statistics and functional analysis of m6A sites indifferent cardiac pathological states

**FIGURE 2:**
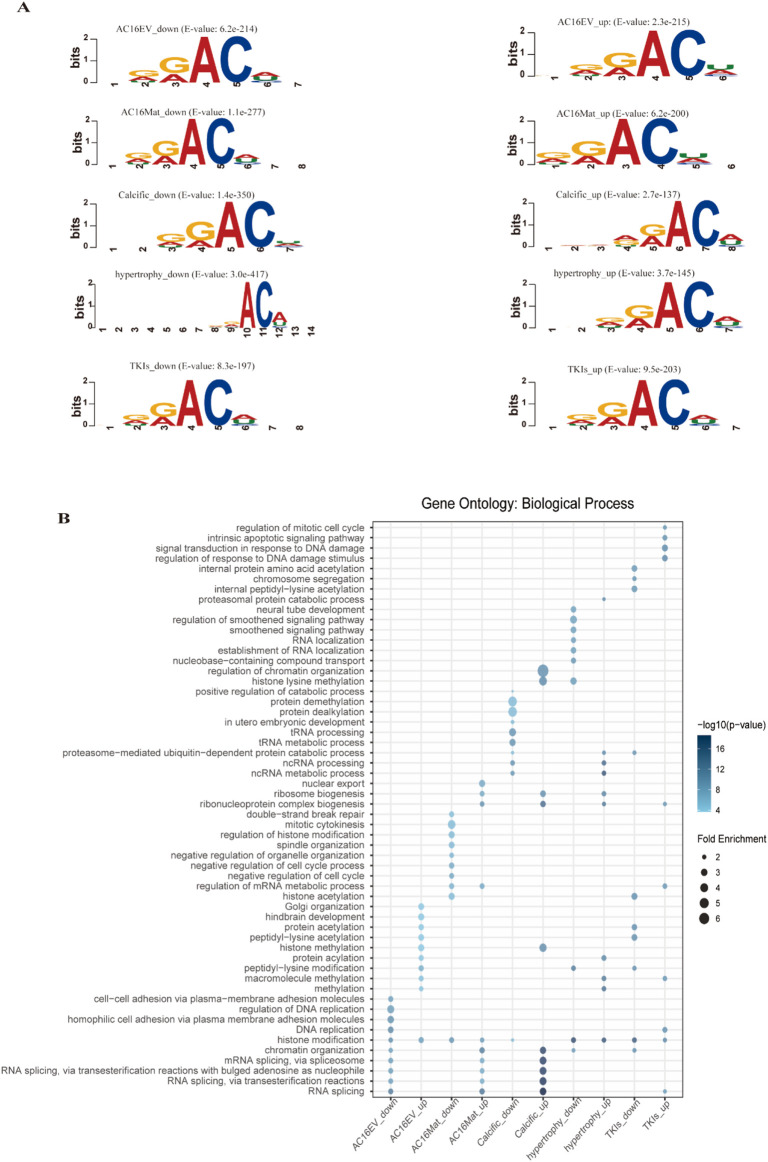
**(A)** The major motifs of Sequences with abnormal m6A modification in five cardiac pathological states using MEME Suite. **(B)** GO enrichment analysis of two abnormal modifications (upregulation and downregulation) of m6A in each cardiac pathological state.

Recent studies have demonstrated that sequence-derived features are reliable and effective in capturing the inherent specificity of target sequences. Therefore, we explored six different encoding methods to compare their effectiveness in predicting m6A modification specificity in different cardiac pathological states. In addition, we performed motif analysis of abnormal m6A modification sequences from five cardiac pathological states using MEME Suite (Bailey and Elkan, 1994). The “RRAC” motif is frequently observed in sequences exhibiting abnormal m6A modification in five distinct cardiac pathological states (Figure 2A). Consequently, it can be deduced that cardiac diseases resulting from aberrant m6A modification may be driven by shared factors.

To explore the correlation between m6A modification sequences, m6A modification and biological functions, we performed Gene Ontology (GO) enrichment analysis on https://www.bioinformatics.com.cn (last accessed on 10 December 2024), an online platform for data analysis (Tang et al., 2023). This approach enabled us to identify the biological processes associated with cardiac pathological states and reveal their potential roles in cellular functions. As illustrated in Figure 2B, the top 10 GO biological process terms associated with the two m6A abnormalities in each cardiac pathological state are shown. We can observe that different biological processes are significantly enriched in the two types of m6A abnormal modifications under various cardiac pathological conditions. For example, evodiamine-induced cardiac toxicity caused by abnormal downregulation of m6A is associated with RNA splicing and DNA replication; whereas m6A upregulation makes evodiamine-induced cardiac toxicity more closely related to histone modification and peptidyl-lysine modification. Similarly, matrine-induced cardiac toxicity caused by abnormal downregulation of m6A is associated with histone modification and negative regulation of the cell cycle, while abnormal upregulation of m6A makes matrine-induced cardiac toxicity more closely related to RNA splicing and chromatin organization. For heart calcification, the biological processes associated with abnormal m6A downregulation are closely linked to tRNA metabolic processes and tRNA processing, while abnormal m6A upregulation is associated with RNA splicing and RNA splicing via transesterification reactions. In studies on cardiac hypertrophy, biological processes induced by abnormal downregulation of m6A primarily involve histone modification and peptidyl-lysine modification, while abnormal upregulation of m6A is associated with ncRNA metabolic processes and histone modification. Finally, TKI-induced cardiotoxicity caused by abnormal downregulation of m6A is associated with histone modification and peptidyl-lysine modification, while abnormal upregulation of m6A is more closely related to regulation of response to DNA damage stimulus and histone modification.

### 3.2 Performance of different length windows

**FIGURE 3:**
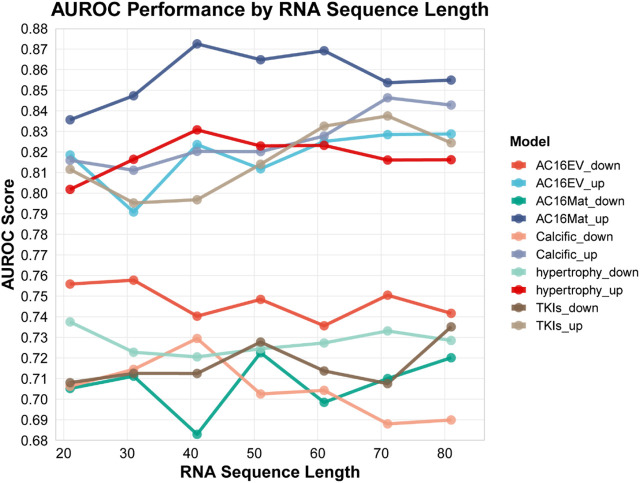
Prediction performance with different window length (Supplementary Table S2).

The amount of sequence information captured varies with the length of the input sequence window, and the selection of window size has a direct impact on the performance of the predictive models (Chen et al., 2021; Chen et al., 2015). To ensure optimal model performance, we systematically evaluated input sequences of varying lengths (21, 31, 41, 51, 61,71, and 81 nt), each centered on the m6A modification site (Figure 3). In the full transcriptome model, as the sequence length increased, the predictive performance for different cardiac pathologies also improved, as evidenced by the increase in the AUROC score. However, beyond a certain length, further increases in the sequence length lead to a decline or stabilization of the predictive performance. Based on these observations, we selected the optimal nucleotide sequence length for each prediction model (based on AUROC scores) to generate RNA sequence features for different pathological states. For AC16EV_down, AC16EV_up, AC16Mat_down, AC16Mat_up, Calcific_down, Calcific_up, hypertrophy_down, hypertrophy_up, TKIs_down, and TKIs_up, we selected sequence lengths of 31 nt, 41 nt, 51 nt, 41 nt, 41 nt, 71 nt, 21 nt, 41 nt, 81 nt, and 71 nt, respectively, to generate RNA sequence features with abnormal m6A modifications under different pathological conditions for the model training.

### 3.3 Performance of different feature combinations

**FIGURE 4:**
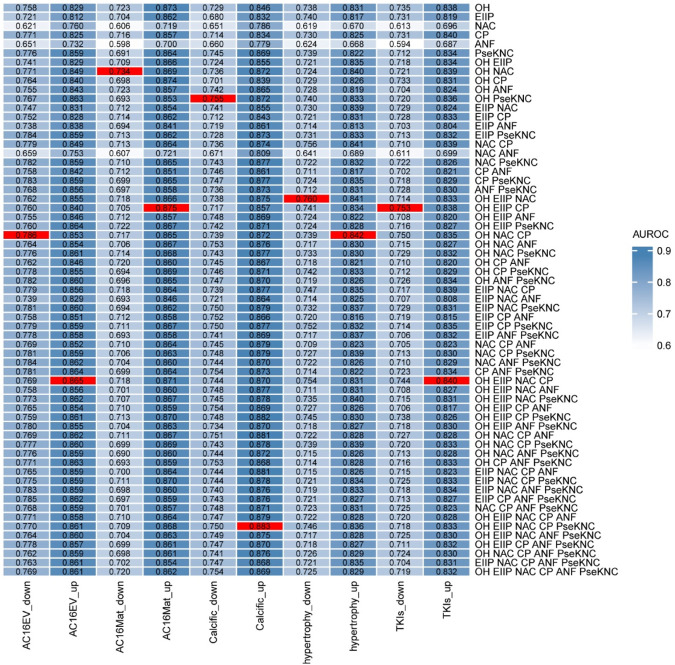
Comparison of different combinations of features. AUROC values of all combinations on independent dataset were evaluated (Supplementary Table S3).

We trained the prediction models using m6A sites that were relatively upregulated or downregulated in each cardiac pathological condition and validated their performance on independent test sets. The optimal feature combinations were identified by calculating the AUROC values across the models. We considered all possible feature combinations derived from the six encoding schemes, including single-feature sets for each pathological state. The model performance (AUROC) for various feature combinations was evaluated, and the results showed that different models excelled when paired with different feature sets. Consequently, for each model, we selected the most suitable feature combination to establish the initial m6AHD (Figure 4).

### 3.4 Algorisms and evaluation

**FIGURE 5:**
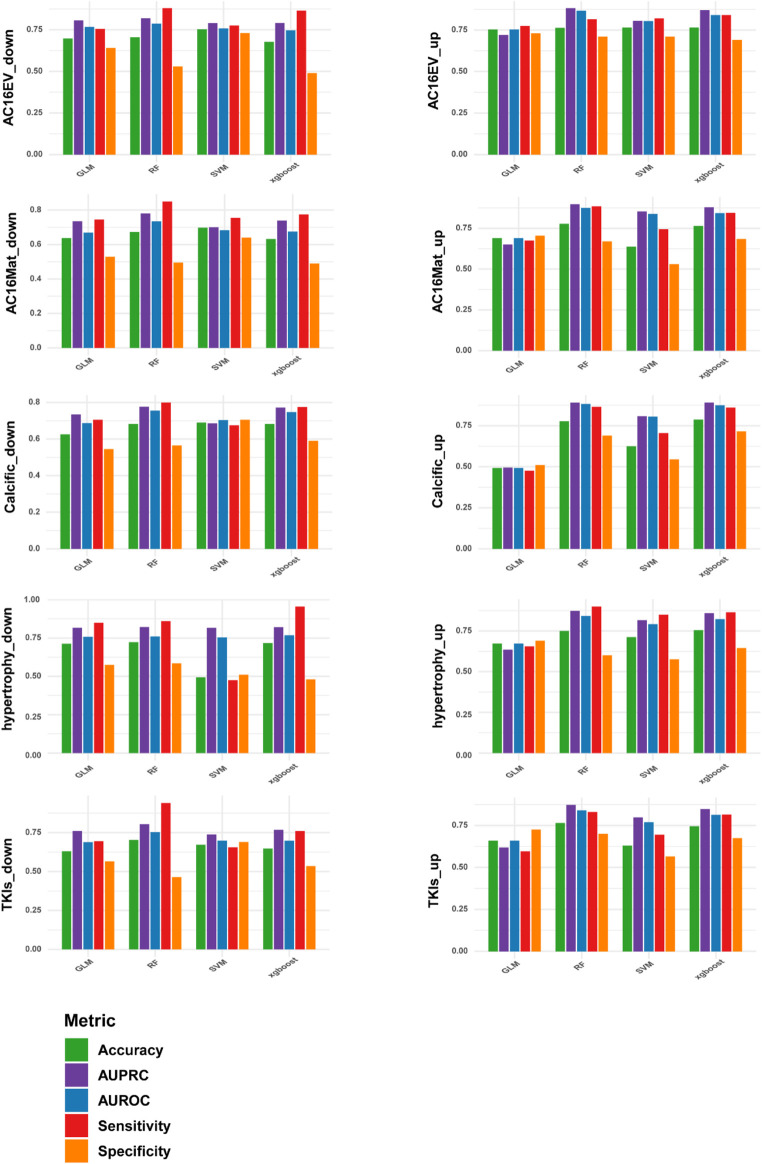
Performance comparison of different machine learning algorithms. Four commonly used machine learning algorithms are compared: SVM, RF, GLM, XGBoost. And AUROC, Acc, Sn, Sp are evaluated, and finally RF is chosen as the model.

Many machine learning algorithms have gained widespread recognition and application in the field of RNA modification prediction because of their excellent predictive accuracy and generalization capabilities (Chen et al., 2021; Chen et al., 2019a; Chen et al., 2019b; Liu et al., 2020b; Huang et al., 2018). To investigate which machine learning algorithm is more suitable for our project to predict different cardiac pathological states, we selected four algorithms—Support Vector Machine (SVM), Random Forest (RF), Generalized Linear Model (GLM), and efficient extreme gradient boosting (XGboost)—for a systematic comparison (Figure 5). We primarily evaluated the performance of the predictive models by calculating the area under the receiver operating characteristic curve (AUROC) on an independent test dataset. We also examined accuracy, sensitivity and specificity. Additionally, we assessed the area under the precision-recall curve (AUPRC) to validate model robustness. In line with the AUROC results, the Random Forest algorithm achieved the highest AUPRC scores for most pathological conditions, outperforming Support Vector Machines (SVMs), Generalised Linear Models (GLMs) and XGBoost. This suggests that the RF-based m6AHD framework strikes an excellent balance between precision and recall, effectively reducing the false positive rate. In summary, the Random Forest algorithm demonstrated the most stable and optimal performance when optimised sequence lengths and feature combinations were employed.

### 3.5 Parameter optimization of the RF model

The parameter settings of the Random Forest model significantly influenced its predictive performance. Two key hyperparameters commonly adjusted are the number of trees (ntree) and the number of features considered at each split (mtry). The ntree parameter specifies the number of decision trees in the ensemble; more trees typically improve stability and reduce variance, although beyond a certain point, the gains diminish and computation becomes inefficient. In contrast, mtry controls the number of candidate predictors randomly selected at each node; lower values increase the diversity among trees and help prevent correlation (thus reducing variance), while higher values allow stronger predictors to be consistently chosen, potentially improving accuracy but risking overfitting. Thus, the careful tuning of ntree and mtry is essential to strike an optimal balance between bias, variance, and computational cost in Random Forest modeling.

**FIGURE 6:**
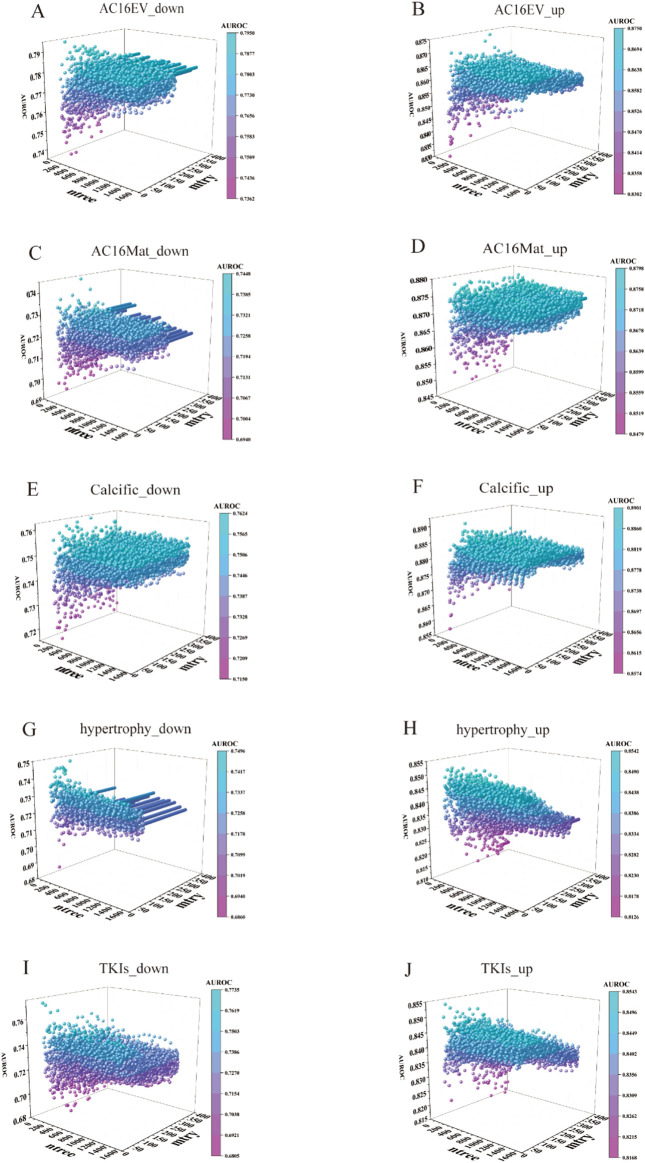
RF model tuning parameters on an independent set. **(A–J)** Adjust the parameters of the RF model, with the x-axis representing the ntree values, and the y-axis representing the mtry values, with the z-axis also indicating the corresponding AUROC values.

**FIGURE 7:**
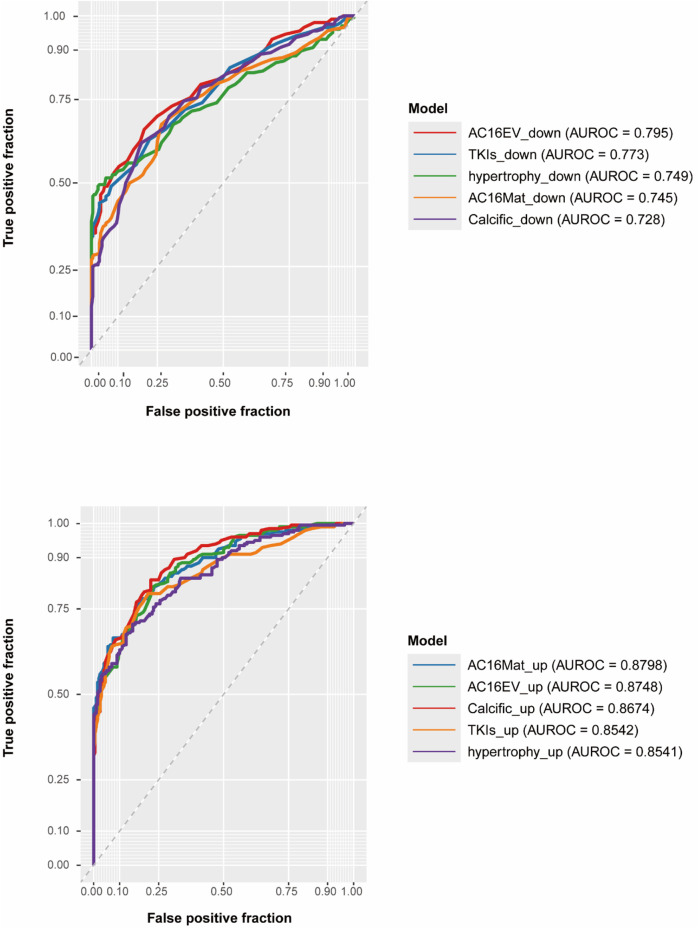
The ROC curves and AUROC values of independent dataset for final model.

In this study, we combined all parameter combinations for the ntree parameter ranging from 200 to 1,400 (in increments of 100) and the mtry parameter ranging from 50 to 100 (in increments of 1), and evaluated the predictive performance of the Random Forest models by calculating AUROC values (Figure 6). Based on the resulting AUROC values, the most appropriate ntree–mtry combination was determined. As a result, we finally determined the optimal predictive model for abnormal m6A modification for each cardiac pathological condition. The AUROC scores for the following model were determined: AC16EV_down, AC16EV_up, AC16Mat_down, AC16Mat_up, Calcific_down, Calcific_up, hypertrophy_down, hypertrophy_up, TKIs_down, and TKIs_up. The AUROC scores were found to be 0.795, 0.875, 0.745, 0.880, 0.728, 0.867, 0.749, 0.854, 0.773 and 0.854, respectively (Figure 7).

### 3.6 Global model interpretability

**FIGURE 8:**
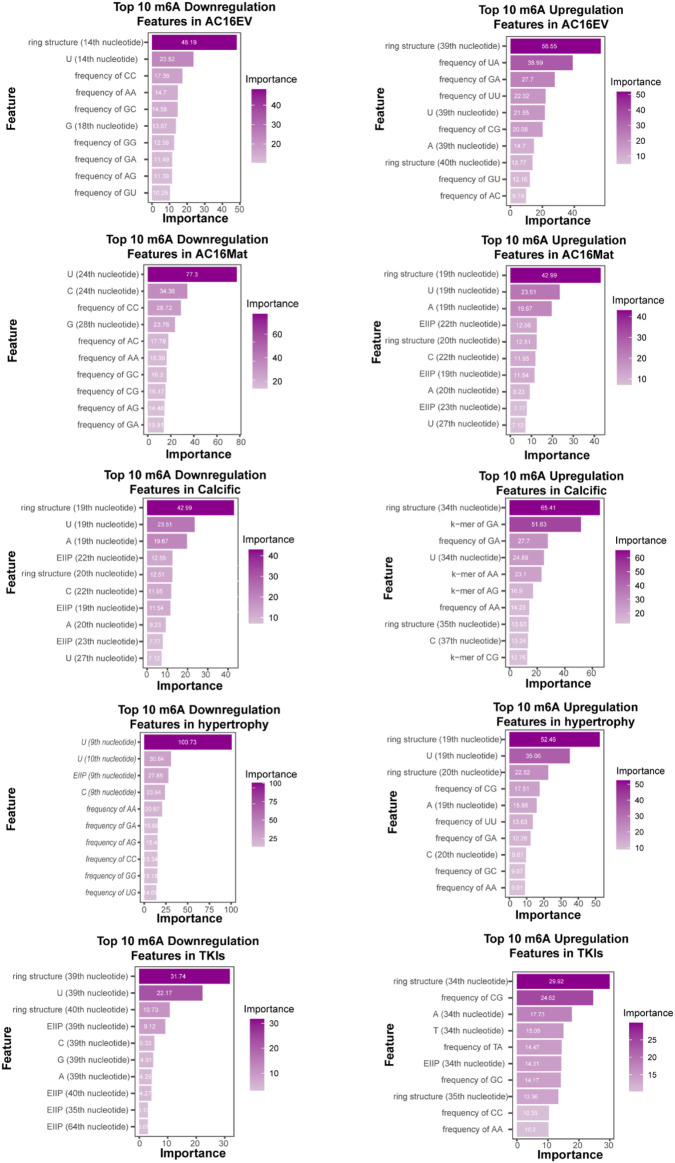
The Mean Decrease Gini rankings for the top 10 most important features in each model.

We evaluated the contribution of each feature to the overall classification task in the final model established based on the Mean Decrease Gini assessment inherent in the random forest model. As shown in the Figure 8, we present a bar chart of the Mean Decrease Gini rankings for the top 10 most important features in each model. It can be observed that the feature indicating whether a nucleotide has a double-ring structure consistently ranks highly in terms of feature importance across multiple models. This suggests that this structural feature plays a critical role in model predictions, indicating that purine nucleotides may frequently participate in m6A modification in RNA, potentially contributing to heart-related diseases.

### 3.7 Model cross-validation prediction

**FIGURE 9:**
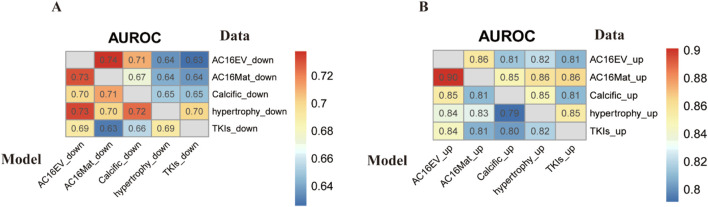
Cross-prediction of predictive models and data on m6A sites in different cardiac pathological states. The values represent the predictive efficacy in terms of AUROC values. The values were visualized by R package, pheatmap. **(A)** Cross-prediction of the predictive model and data for m6A downregulation. **(B)** Cross-prediction of the predictive model and data for m6A upregulation.

Based on the optimized model described above, we estimated the crosstalk between m6A modification patterns in different cardiac pathological states (Figures 9A,B). It can be observed that, irrespective of the status of m6A modification (upregulated or downregulated), the AUROC scores obtained using disparate models on the same dataset are generally similar, indicating the presence of shared m6A modification sites across distinct cardiac pathological states. Consequently, we hypothesise that m6A-modified sequences exhibit analogous characteristics in heart-related diseases, and that the trained models are capable of more effectively capturing the effects of m6A modification on these diseases.

### 3.8 Evaluating on independent dataset

**FIGURE 10:**
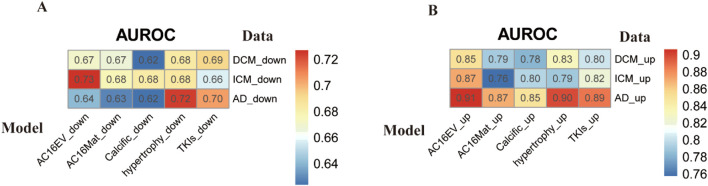
Predictive performance of the m6AHD model for independent datasets. The values in the figure represent the predicted effects expressed as AUROC values. These values are visualized by the R package pheatmap. **(A)** Independent predictions of the prediction model with m6A downregulated data. **(B)** Independent prediction of the prediction model with m6A upregulation data.

To further evaluate the reliability and generalizability of m6AHD, we assessed its performance on independent datasets. Specifically, we selected two datasets from the GEO database encompassing three cardiac pathological conditions—dilated cardiomyopathy, ischemic cardiomyopathy, and aortic dissection—and constructed the corresponding feature profiles. The finalized model was then applied to these independent cohorts for prediction. The results demonstrated that our model could effectively identify other cardiac diseases with favorable generalizability, further confirming the similarity of m6A modification patterns across cardiac disorders (Figures 10A,B). These findings highlight the robustness of our approach and underscore its significance for advancing research on the relationship between m6A modifications and cardiovascular diseases.

### 3.9 Experimental validation of m6A methyltransferase dysregulation in evodiamine-induced cardiotoxicity

**FIGURE 11:**
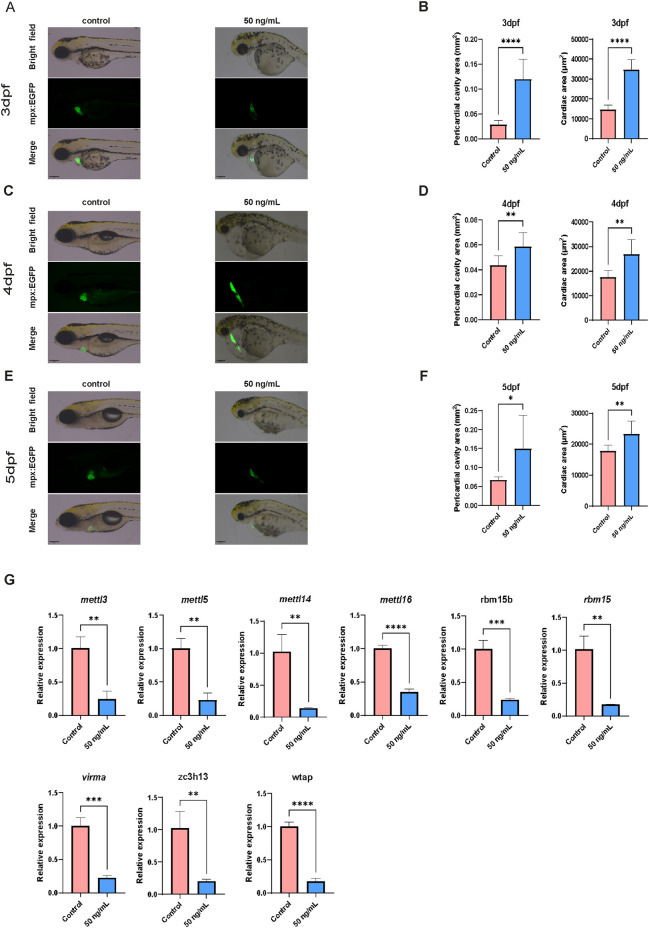
Validation of cardiotoxicity and m6A regulator expression in a zebrafish model. **(A,C,E)** Representative bright-field and fluorescence images of zebrafish larvae at 3 dpf, 4 dpf, and 5 dpf treated with vehicle (Control) or 50 ng/mL Evodiamine. **(B,D,F)** Quantification of the pericardial cavity area and cardiac area at 3, 4, and 5 dpf. Data are presented as mean ± SD. (*P < 0.05, **P < 0.01, ***P < 0.001, ****P < 0.0001). **(G)** Relative mRNA expression levels of m6A methyltransferases (mettl3, mettl5, mettl14, mettl16) and associated regulatory factors (rbm15b, rbm15, virma, zc3h13, wtap) measured by RT-qPCR.

To verify the association between cardiac pathology and the m6A modification machinery, as inferred by our computational framework, we established a model of evodiamine-induced cardiotoxicity in zebrafish. As shown in Figures 11A–F, exposure to 50 ng/mL Evodiamine resulted in severe cardiac morphological defects across 3, 4, and 5 dpf. The treated group exhibited significantly enlarged pericardial cavity areas and cardiac areas compared to the control group (P < 0.05), indicating successful induction of heart failure-like phenotypes and edema.

To determine if this cardiotoxicity involves the dysregulation of m6A regulatory enzymes, we examined the expression of key m6A methyltransferases. RT-qPCR analysis (Figure 11G) revealed a widespread and significant downregulation of core component genes of the m6A methyltransferase complex and regulatory subunits in the 50 ng/mL group (P < 0.01).

It is worth noting that in our preliminary investigation, we exposed zebrafish to a lower concentration of Evodiamine. As demonstrated in Supplementary Figure S1, the low-dose group did not exhibit significant cardiac malformations or edema compared to controls. Correspondingly, RT-qPCR analysis demonstrated that the expression levels of m6A writers in this phenotypically normal group were preserved, showing no significant difference from the control group.

The collective *in vivo* results are consistent with the premise of the predictive model. The observation that significant suppression of m6A writers occurred specifically in the high-dose group with severe toxicity–but not in the low-dose group with preserved morphology–strongly suggests that the extent of aberrant m6A modification regulation is closely correlated with the severity of cardiac pathology. The data suggests that Evodiamine-induced cardiotoxicity may be driven by a threshold-dependent collapse of the m6A methylation machinery, leading to a global hypomethylation state that is essential for the disease phenotype.

## 4 Conclusion

In this study, we proposed a whole-transcriptome prediction model based on the RF algorithm to predict five cardiac pathological states—evodiamine-induced cardiotoxicity, matrine-induced cardiotoxicity, TKI-induced cardiotoxicity, hypertrophy, and heart calcification using m6A modification information. To pursue the most effective feature combination, we selected six sequences and trained the model by combining the best encoding methods for each feature based on the model with the best predictive performance under different feature combinations. Our framework achieved high performance on an independent test set. After comparing different machine learning algorithms, we ultimately selected RF as the model. We then adjusted the parameters of the RF model, compared the predictive performances of different parameter combinations, and selected the optimal predictive parameters to construct the final predictive model. We also conducted a Mean Decrease Gini assessment of the global importance of each feature in the model based on the random forest model, speculating that m6A modification tendencies appear in regions rich in purine nucleotides, thereby leading to various cardiac pathological states.

Our computational analysis suggested a strong association between cardiac pathologies and the dysregulation of m6A modification enzymes. This hypothesis was biologically corroborated by our subsequent *in vivo* experiments using an Evodiamine-induced zebrafish model. We observed that Evodiamine treatment not only induced severe pericardial edema but also significantly suppressed the expression of the core m6A methyltransferase complex, underscoring the critical role of m6A homeostasis in cardiomyocyte function. These findings indicate that the ‘abnormal m6A sites’ predicted by m6AHD in the white-barked lobelia alkaloid dataset likely stem from defects in methylation writing capacity. Notably, our pattern analysis revealed significant enrichment of the ‘RRAC’ consensus sequence among these anomalous sites (Figure 1). Given that this motif constitutes a classic substrate for the m6A methyltransferase complex, the observed global downregulation of methyltransferases provides a direct mechanistic explanation for the model-predicted site-specific hypomethylation. This validates the biological relevance of the model’s feature selection, particularly demonstrating that targets containing RRACH sequences exhibit differential expression when enzymatic activity is severely compromised.

Recently, tissue-specific m6A prediction tools have been developed for multiple organ systems (Song et al., 2023a; Jiang et al., 2024; Dao et al., 2020; Liu K. et al., 2020; Xia et al., 2024). However, the cardiovascular system possesses unique electrophysiological and contractile properties governed by distinct gene regulatory networks. As the first framework specifically trained on multi-condition cardiac datasets (toxicity, hypertrophy, calcification), m6AHD captures the unique epigenomic features of cardiac disease that may be overlooked by general models or other tissue-specific approaches.

Although this study has made progress in utilizing m6A modifications to predict different cardiac pathological states, we acknowledge several limitations. First, it is important to note that while this study relies on MeRIP-seq data, the field of epitranscriptomics is rapidly evolving towards direct RNA sequencing (DRS) using the Nanopore platform. Unlike antibody-based detection methods, DRS directly analyses RNA strands by monitoring the specific interruption of ionic currents triggered when molecules pass through nanopores. This mechanism eliminates biases introduced by reverse transcription or PCR amplification and enables the identification of modification stoichiometry and allele-specific patterns at single-nucleotide resolution (Zhang et al., 2023; Wu et al., 2024; Guo et al., 2025; Kovaka et al., 2025; Alagna et al., 2025; Zhang et al., 2025). Although MeRIP-seq remains a cost-effective approach for whole-transcriptome screening, we anticipate that future iterations of the m6AHD framework will integrate DRS data to further enhance predictive accuracy and resolution. Second, our current model relies on bulk MeRIP-seq data obtained from complex cardiac tissues containing a mixture of cardiomyocytes, fibroblasts, and immune cells. Consequently, the predicted abnormal m6A patterns represent aggregated signals that may partially reflect variations in cell-type composition. Future work should utilise picoMeRIP-seq to disentangle these signals and precisely localise cardiomyocyte-specific alterations (Li et al., 2024). Thirdly, while sequence-derived features demonstrate significant predictive ability, RNA methylation is also greatly affected by RNA secondary structure and its interactions with RNA-binding proteins. Future iterations of the m6AHD model could incorporate high-throughput RNA structural probe data, such as *in vivo* click-selective 2′-hydroxyacylation with spectral analysis (icSHAPE) (Spitale et al., 2015), to capture the structural accessibility of RNA regions. Furthermore, incorporating tissue-specific CLIP-seq (cross-linking immunoprecipitation sequencing) data would allow the model to consider the binding patterns of relevant RNA-binding proteins (RBPs), which may compete with or facilitate methylation mechanisms. Together, integrating these multidimensional features could further enhance the biological interpretability and predictive accuracy of the m6AHD model. Finally, while current random forest models based on manually designed features have demonstrated robust performance, the rapid advancement of deep learning technologies offers significant potential for further improvement. Deep learning architectures, such as convolutional neural networks (CNNs) and Transformer-based models, can automatically learn high-level abstract representations from raw sequence data and model long-range dependencies between nucleotides. Future iterations of m6AHD could use these advanced algorithms to identify more subtle and complex methylation patterns that traditional machine learning approaches might miss.

## Data Availability

The original contributions presented in the study are publicly available. The project code is deposited at https://github.com/Jack-Neo/m6AHD. The dataset GSE227247 is publicly available at the NCBI Gene Expression Omnibus (https://www.ncbi.nlm.nih.gov/geo/).
