# Supplementary figures and images for: m6AHD: a new framework for identifying abnormal N6-methyladenosine (m6A) in heart diseases based on sequencing features
Source: Front Genet. 2026 Feb 25;17:1776616. doi: 10.3389/fgene.2026.1776616 (PMC12975140; doi:10.3389/fgene.2026.1776616)

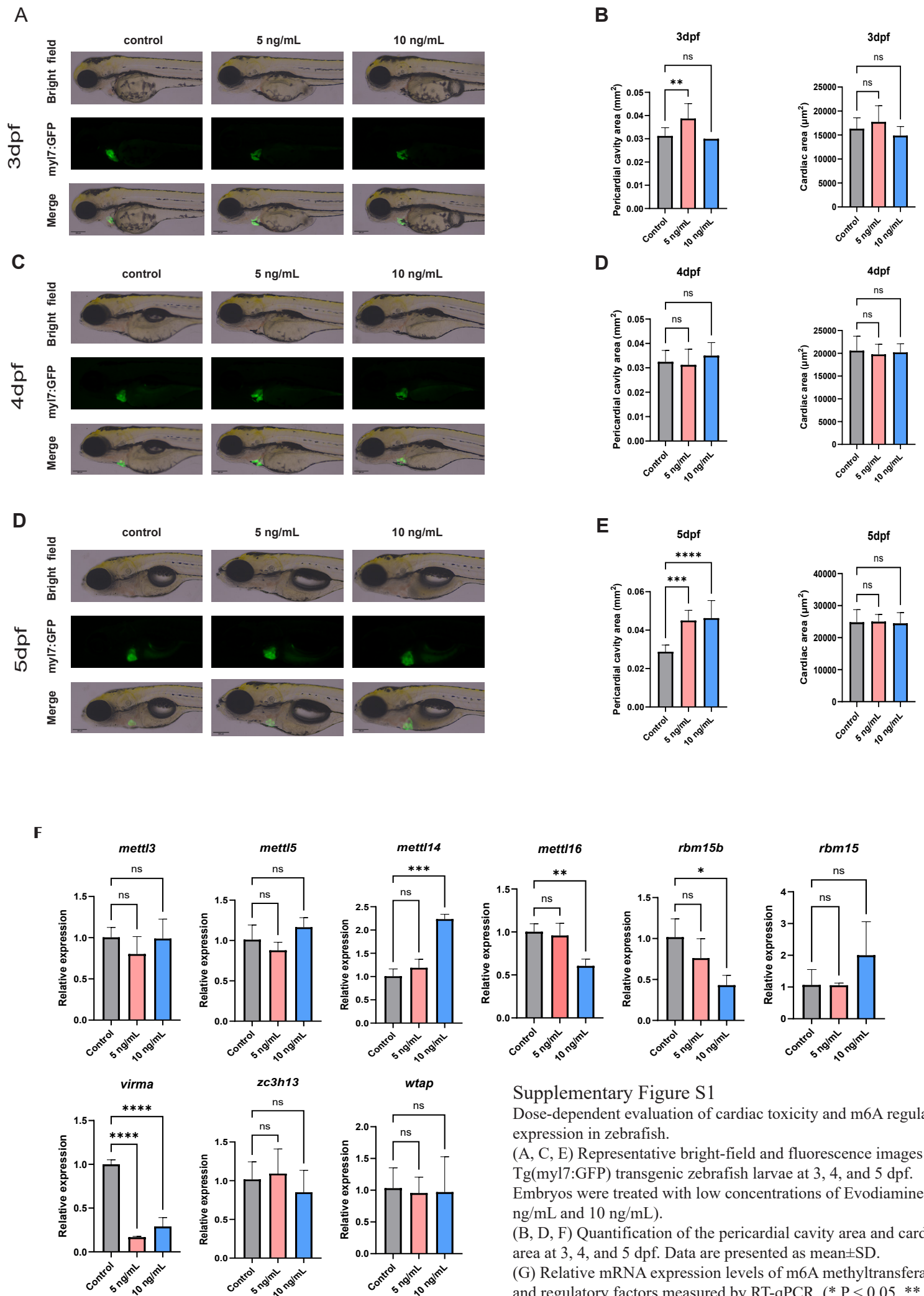

Supplement: Supplementary file 3 [file DataSheet1.pdf]
